# Supplementary material for: A Real-World Data Observational Analysis of the Impact of Liposomal Amphotericin B on Renal Function Using Machine Learning in Critically Ill Patients
Source: Antibiotics (Basel). 2024 Aug 12;13(8):760. doi: 10.3390/antibiotics13080760 (PMC11350854; doi:10.3390/antibiotics13080760)
Supplement: Supplementary file 1 [file antibiotics-13-00760-s001.zip › antibiotics-3124650-supplemenatry.pdf]

Supplementary Materials

Table S1. Coded entry diagnoses of the 84 patients eligible for the study.

Codified diagnosis	n (%)
1.-COVID-19	18 (21.4)
2.- Ventilator associated pneumonia	14 (16.6)
3.- Community-acquired pneumonia	12 (14.3)
4.- Sepsis and septic shock	9 (10.6)
5.- Human immunodeficiency virus (HIV)	7 (8.3)
6.- Pulmonary aspergillosis	5 (6.0)
7.-Abdominal sepsis	4 (4.8)
8.- Brain abscess	2 (2.4)
9.- Retropharyngeal abscess	2 (2.4)
10.- Necrotising fasciitis	2 (2.4)
11.- Acute myeloid leukaemia	2 (2.4)
12.- Lymphoma	2 (2.4)
13.- Rhinocerebral mucormycosis	2 (2.4)
14.- Influenza A (H1N1)pdm09 pneumonia	2 (2.4)
15.- Liver Abscess	1(1.27

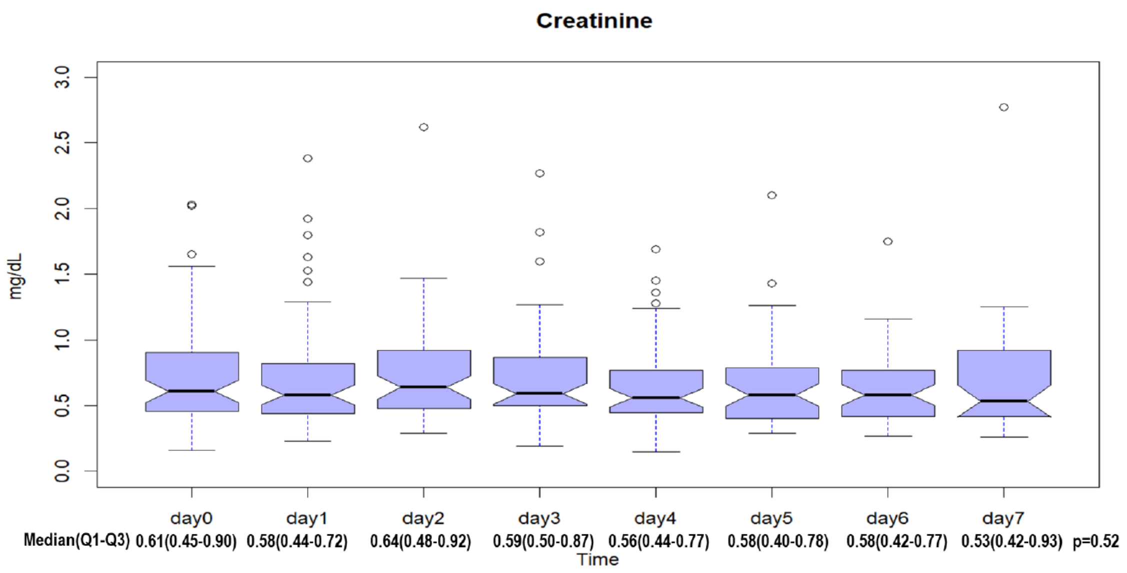

Figure S1. Creatinine levels during observation period in 67 patients included.

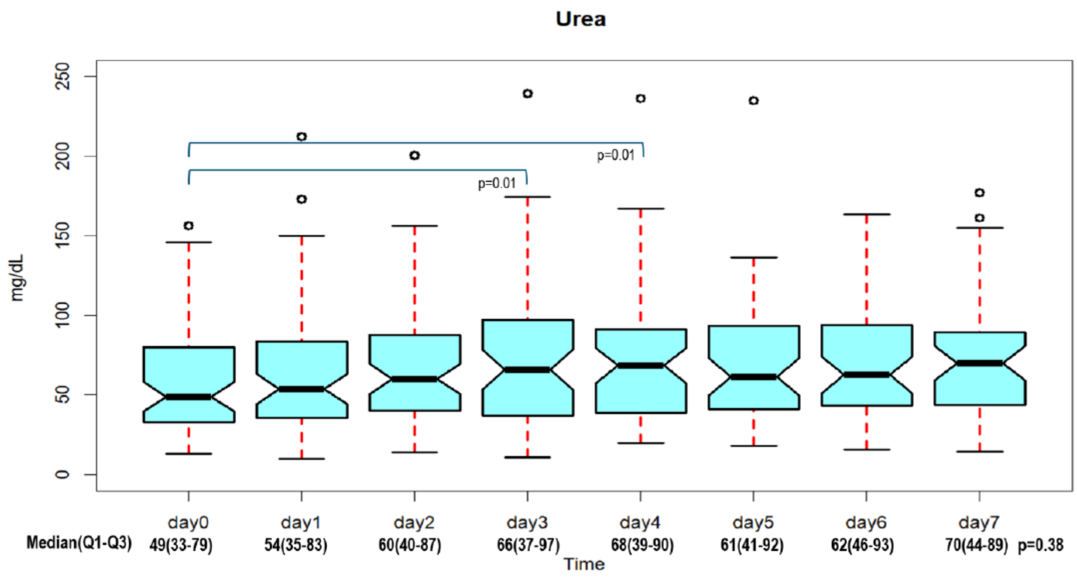

Figure S2. Urea levels during observation period in 67 patients included.

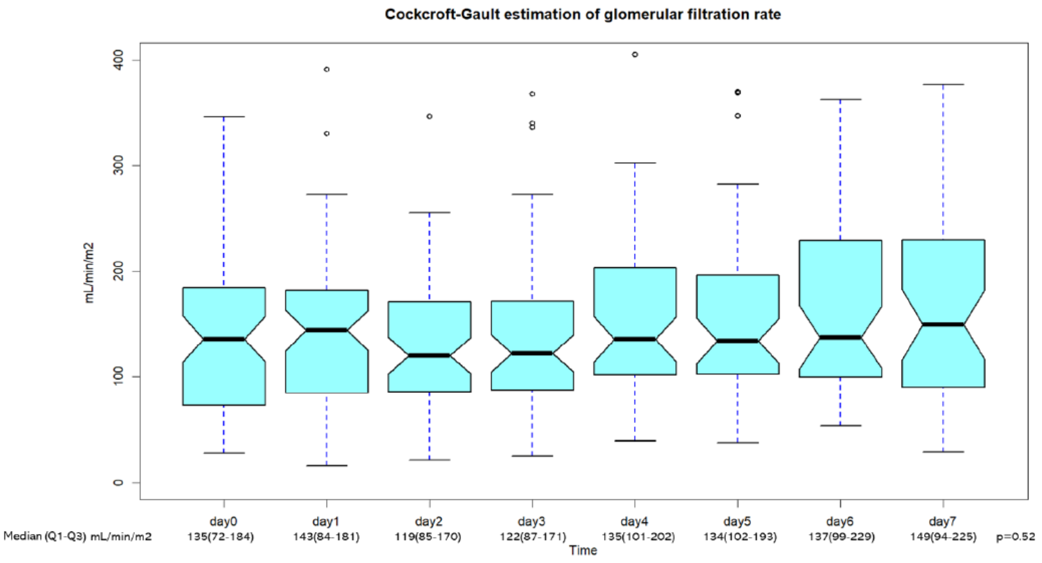

Figure S3. Cockcroft-Gault estimation of glomerular filtration rate in whole population.

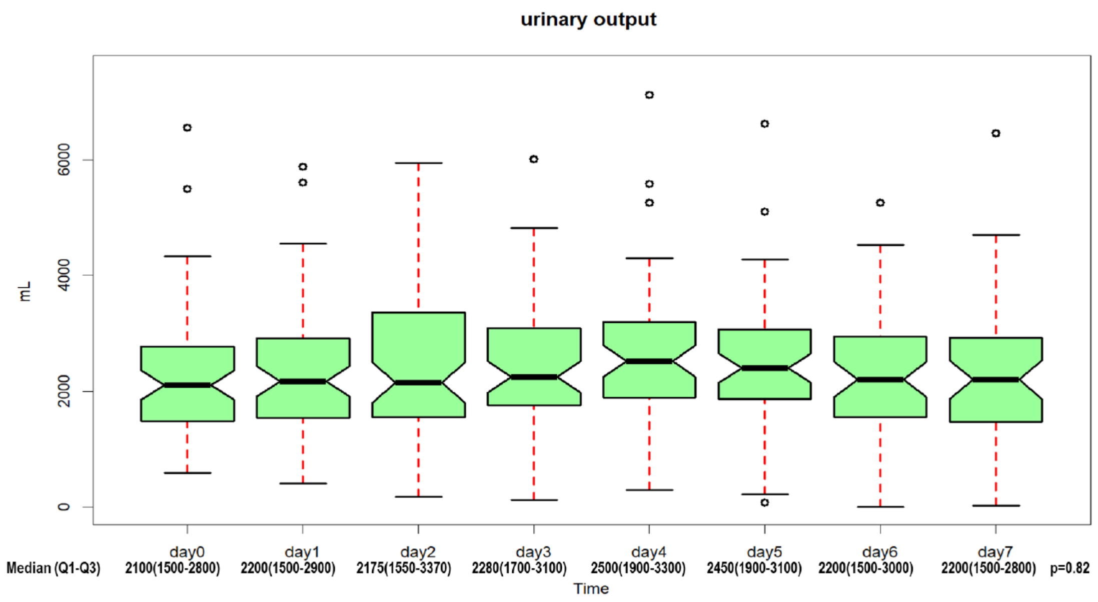

Figure S4. Urinary output during observation period in 67 patients included.

Statistical analysis

Our analysis plan is based on the following five steps:

Step 1: The behavior of serum creatinine and urea levels as well as urinary volume during the study period (day 1 to day 7) was analyzed. Categorical variables are presented as number and percentage (%), and continuous variables as median and interquartile range (Q1-Q3). To analyze differences between groups, the Mann-Whitney U-test (continuous) and Chi-square (dichotomous) were used. Temporal differences between means were determined by Analysis of Variance (ANOVA) and paired ANOVA with Bonferroni correction.

Step 2: The linear association between variables of interest (creatinine, urea and total L-AmB dose administered was determined by obtaining Spearman (Rho) correlation coefficient due to the non-parametric distribution of the data.

Step 3: The incidence of AKI on day 3 of L-AmB administration was determined in the general population according to the definition considered. A bivariate comparison was made between the groups with and without AKI on day 3 of observation.

Step 4: The impact of the different variables on the development of AKI was established by multivariate analysis (multiple logistic regression). The result is shown as Odds Ratio (OR) with a 95% confidence interval (95% CI). Values below 0.05 were considered significant. The assessment of model fit was performed using the Akaike's information criterion (AIC).

Because no significant differences were observed between patients with and without AKI. The logistic regression model (glm) was performed considering the presence of AKI at day 3 as the dependent variable (yes=1/no=0). All variables at day 3 were included as independent variables. The inclusion of quantitative variables required the presence of at least 10 patients with the event. When running the model with all variables a warning message is observed: “Warning: glm.fit: fitted probabilities numerically 0 or 1 occurred” and that the model does not achieve significance and the CIs are too large.

```
Number of Fisher Scoring iterations: 15

Waiting for profiling to be done...
Warning: glm.fit: fitted probabilities numerically 0 or 1 occurred
Warning: glm.fit: fitted probabilities numerically 0 or 1 occurred
Warning: glm.fit: fitted probabilities numerically 0 or 1 occurred
Warning: glm.fit: fitted probabilities numerically 0 or 1 occurred
Warning: glm.fit: fitted probabilities numerically 0 or 1 occurred
Warning: glm.fit: fitted probabilities numerically 0 or 1 occurred
Warning: glm.fit: fitted probabilities numerically 0 or 1 occurred
Warning: glm.fit: fitted probabilities numerically 0 or 1 occurred
Warning: glm.fit: fitted probabilities numerically 0 or 1 occurred
Warning: glm.fit: fitted probabilities numerically 0 or 1 occurred
Warning: glm.fit: fitted probabilities numerically 0 or 1 occurred
Warning: glm.fit: fitted probabilities numerically 0 or 1 occurred
Warning: glm.fit: fitted probabilities numerically 0 or 1 occurred
Warning: glm.fit: fitted probabilities numerically 0 or 1 occurred
Warning: glm.fit: fitted probabilities numerically 0 or 1 occurred
Warning: glm.fit: fitted probabilities numerically 0 or 1 occurred
```

	OR	2.5 %	97.5 %	p	
(Intercept)	6.2749e-15	3.4775e-44	2.5893e+09	0.27030	
Male	6.1623e+00	4.3170e-01	1.7535e+02	0.21980	
Age	9.5201e-01	8.5174e-01	1.0447e+00	0.31874	
Weight	9.1285e-01	7.7649e-01	1.0268e+00	0.19413	
L-AmB duration	8.4798e-01	6.2233e-01	1.0199e+00	0.12609	
SOFA at day 3	2.2218e+00	6.7218e-01	1.0453e+01	0.24248	
WBC at day 3	8.6592e-01	5.4002e-01	1.1419e+00	0.44709	
Lymphocytes at day 3	2.4989e+00	9.8237e-02	2.0046e+02	0.60723	
CRP	9.8764e-01	7.8105e-01	1.2121e+00	0.90374	
PCT	9.7874e-01	8.4304e-01	1.1249e+00	0.75404	
Sodium at day 3	1.2131e+00	9.1917e-01	1.7344e+00	0.20784	
Potassium at day 3	1.2104e-01	5.1517e-03	1.9224e+00	0.15123	
Bilirrubin at day 3	7.9647e-01	2.6824e-01	1.7769e+00	0.66335	
Albumin at day 3	2.2165e+04	1.1013e+00	4.1633e+10	0.08737	.
Hemoglobin at day 3	2.3039e-01	3.5907e-02	9.1950e-01	0.05805	.
Creatinine at day 3	2.9759e-01	2.3149e-03	3.3348e+01	0.60067	
Urea at day 3	1.0518e+00	1.0057e+00	1.1174e+00	0.04770	*
Norepinephrine at day 3	8.6945e-04	1.4578e-07	1.7140e-01	0.03805	*
Metamizole	5.9089e+02	7.6029e+00	8.7138e+05	0.02444	*
Furosemide	1.4910e+00	9.5352e-02	3.1233e+01	0.77616	
Amikacin	7.2554e+00	2.2515e-01	2.8344e+02	0.25403	
Foscarnet	2.9376e+04	1.5740e+01	3.0104e+09	0.02477	*
Rifampicin	2.1088e-05	NA	2.3968e+64	0.99642	
Diabetes	2.8090e+01	7.8606e-01	2.5335e+03	0.09645	.
Chronic liver dis.	1.1004e-02	2.1288e-06	5.1952e+00	0.22622	
Hypertension	2.6635e-01	7.0396e-03	6.7396e+00	0.43300	
ID	2.9175e-01	5.4093e-03	9.9589e+00	0.50206	

```
Null deviance:73.660 on 66 degrees of freedom
Residual deviance:40.241 on 40 degrees of freedom
AIC: 94.241
```

The warning means that when R is computing probabilities internally, as part of the fitting process, they sometimes "underflow/overflow" - that is, they're so close to 0 or 1 that they can't be distinguished from them when using R's standard 64-bit floating-point precision (e.g. values less than about 1e-308 or greater than about

1-1e-16). It simply means that one or more observations in the data frame have predicted values indistinguishable from 0 or 1.

The analysis of the residuals confirms that the model does not work properly.

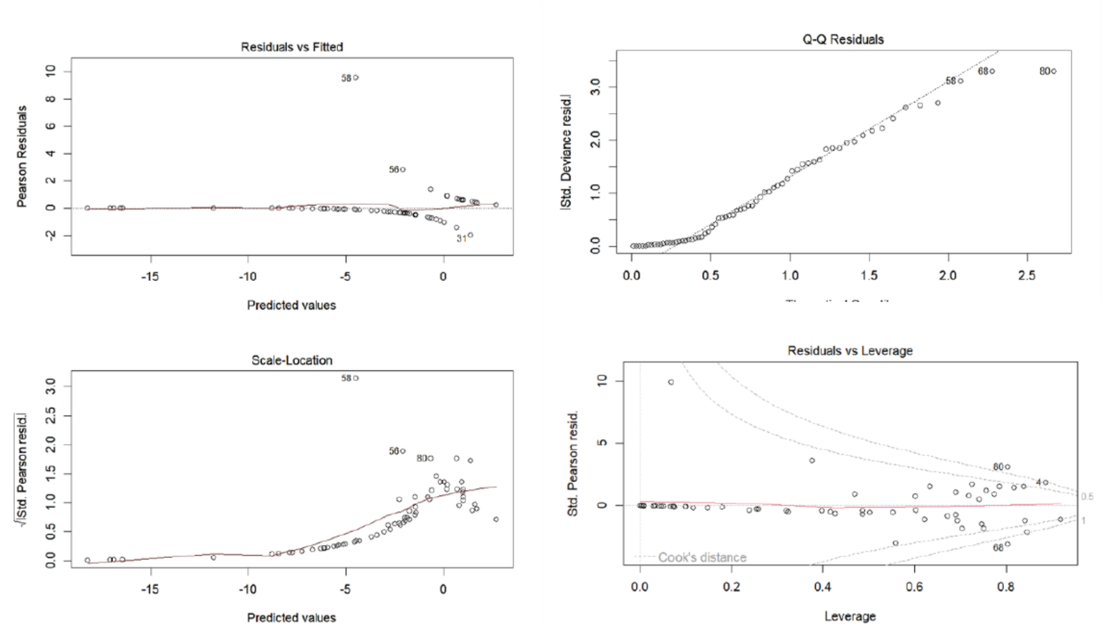

Different models were run with the inclusion and exclusion of variables. The best model is the one shown in Figure S6. However, we did not find any variables independently associated with the development of AKI at day 3. The ORs and confidence intervals of the model can be seen in Table S2.

Table S2. Odds ratio and confidence intervals of the model conducted to assess factors associated with the development of AKI at day 3.

	OR	2.5 %	97.5 %	p
(Intercept)	0.15332980	0.00044161	48.2614	0.5184
L-AmB total dose day 3	1.00012976	0.99846187	1.0016	0.8658
SOFA at day 3	1.15613904	0.81568044	1.6872	0.4225
Age	0.98492004	0.94516368	1.0267	0.4636
Male sex	0.78845860	0.18815492	3.4527	0.7439
Weight	1.03567274	0.98353319	1.0947	0.1924
CRP at day 3	1.01990140	0.94029370	1.1086	0.6337
Potassium at day 3	0.58482582	0.18333950	1.6648	0.3336

The study of the residuals also failed to demonstrate adequate performance of the model.

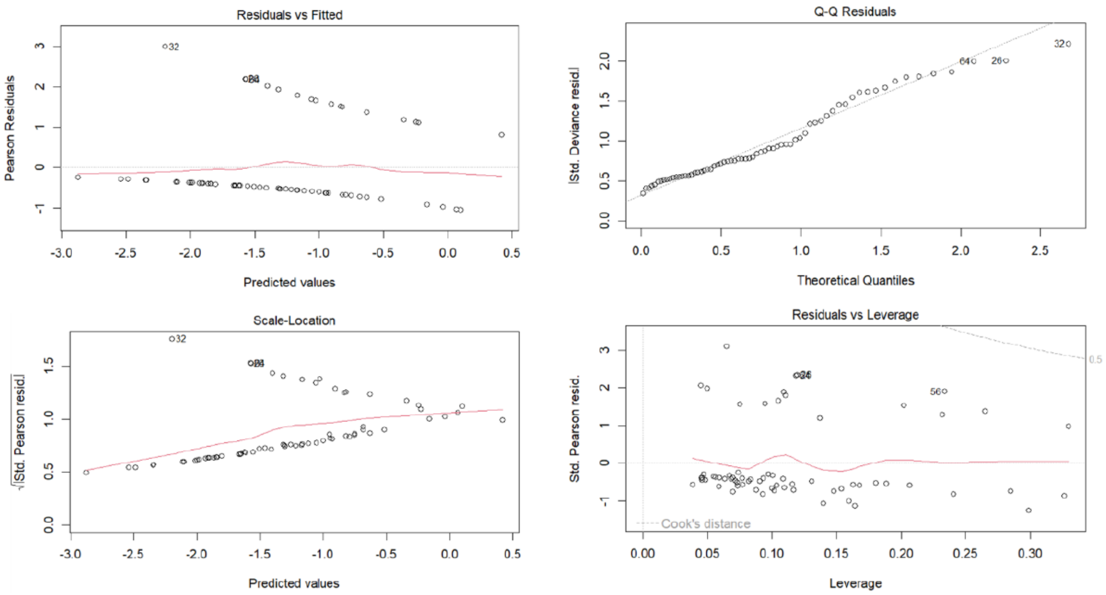

With these findings, it can be hypothesised that linear models cannot explain or cannot find variables associated with the dependent variable (AKI at day 3). For this reason, non-linear models such as Random Forest have been used.

Model Validation

The models have been run to determine which variables are associated with or have an influence on the development of AKI, which is why no internal validation of the linear model has been performed. In fact, we do not intend to make a predictive model or that it should be applied to another population, but only to discover or determine which variables are associated with the development of AKI. On the other hand, the relatively small number of patients included (n=56) makes it impractical to divide it into a development (training) and a validation (test) subgroup.

Due to the instability of linear regression models a non-linear Random Forest (RF) model was developed to study the impact of covariates on the development of AKI. The Random Forest algorithm is a powerful non-linear tree-based learning technique in machine learning. The performance of the RF model was evaluated by the out-of-bag (OOB) error. This method allows measuring the prediction error of random forests, boosted decision trees and other machine learning models using bootstrap aggregation.

The models developed are presented in the main body of the manuscript (Figures 2 and 3) as well as their evaluation using the out-of-bag (OOB) error.

Internal validation of the model has also not been performed for the same reasons as stated for the linear models. These models are not intended to be predictive and have not been developed for application to populations other than the study population. The aim is to discover the contribution of the different variables in the development of AKI.

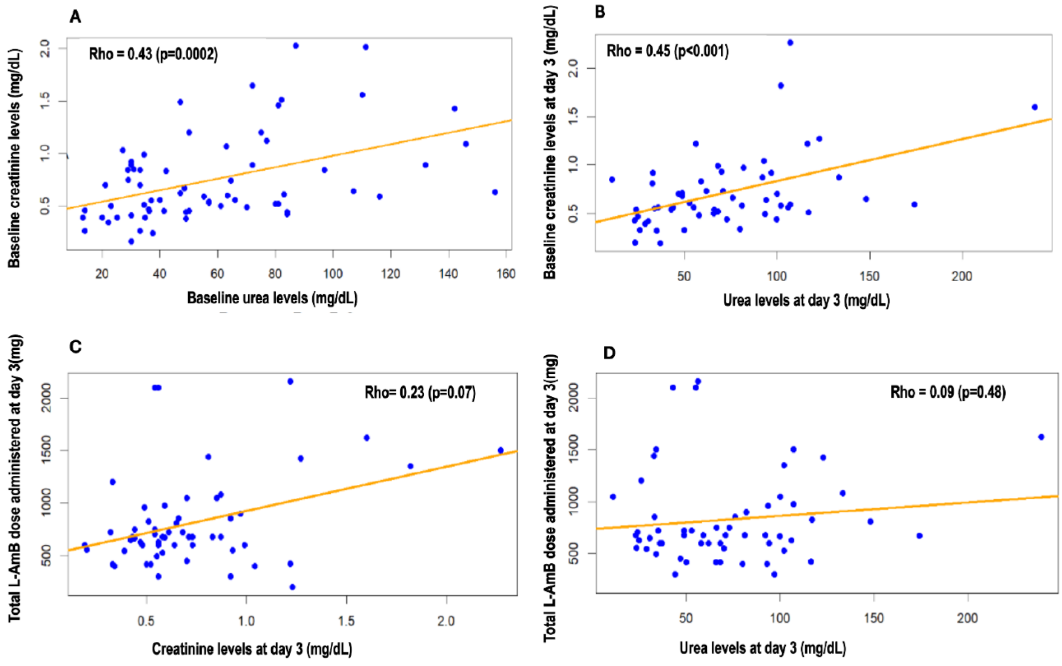

Figure S5. Correlation (Spearman) between creatinine (A) and urea (B) (baseline and at day 3) and creatinine (C) and urea (D) at day 3 with respect to the total dose of liposomal amphotericin (L-AmB) at day 3.

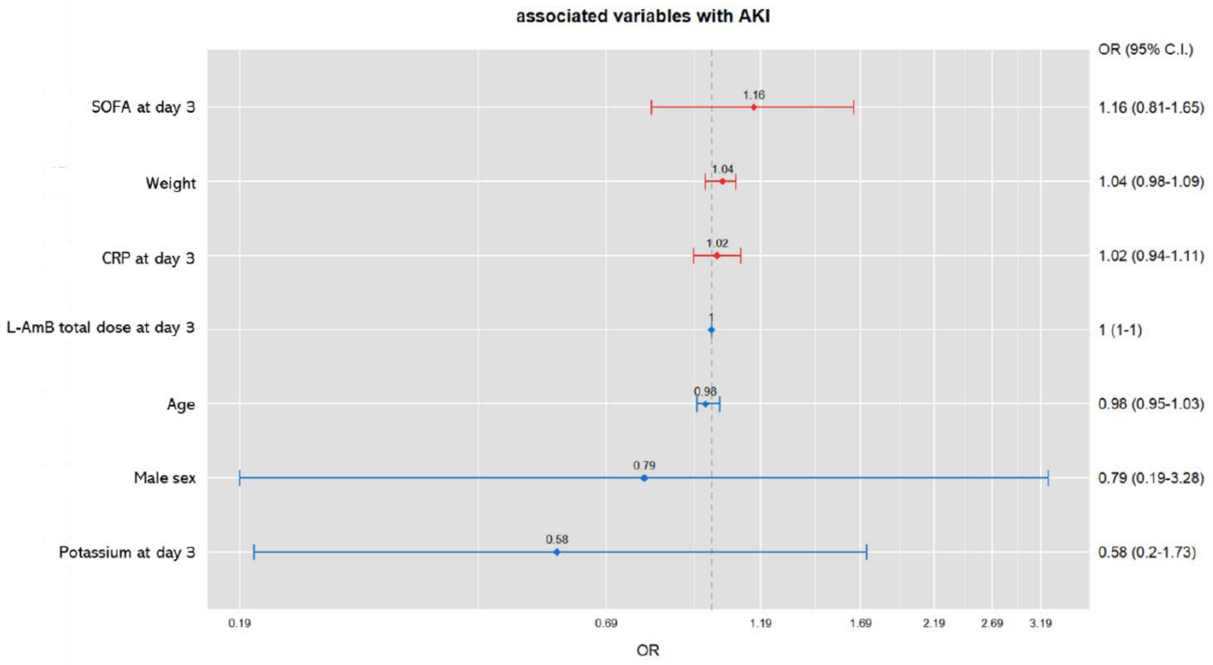

Figure S6. Variables associated with the development of AKI at day 3 of L-AmB administration in whole population (logistic regression analysis) (L-AmB: Liposomal amphotericin B; RCP: reactive C protein; K+: serum potassium).

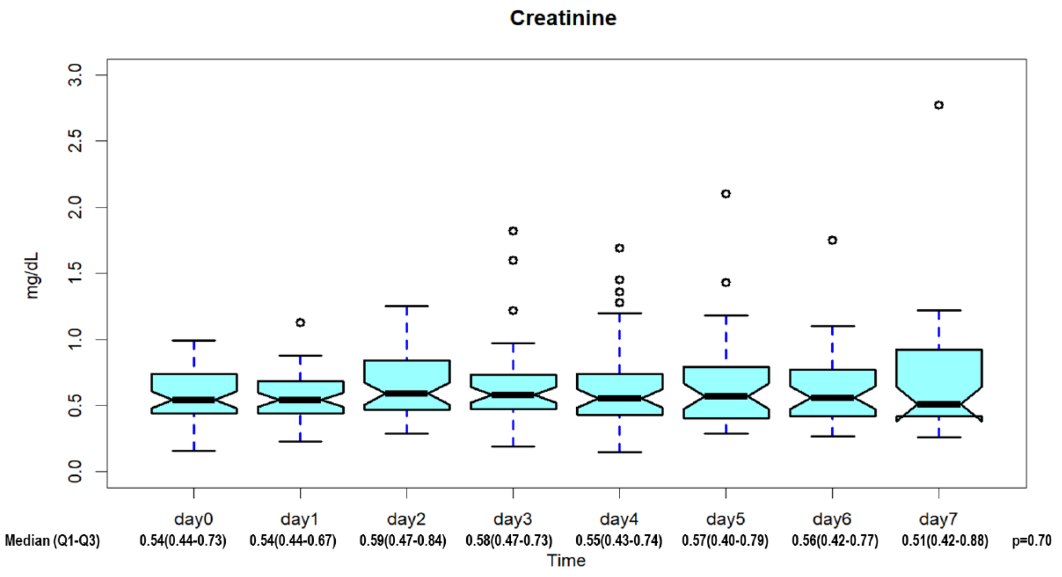

Figure S7. Creatinine levels during observation period in 52 patients with low risk of AKI at baseline (Day 0).

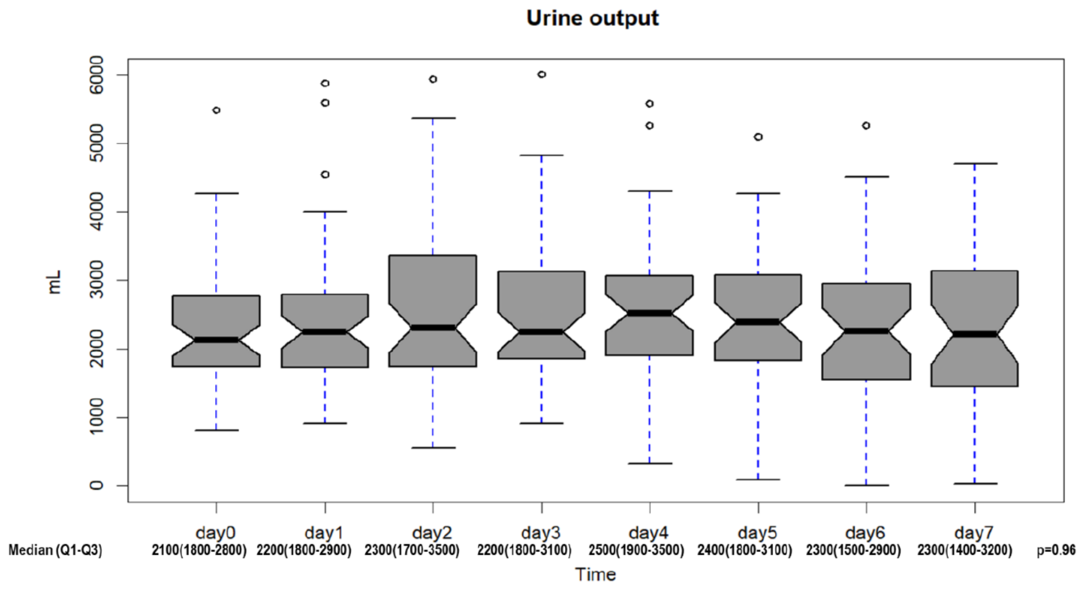

Figure S8. Urine output during observation period in 52 patients with low risk of AKI at baseline (Day 0).

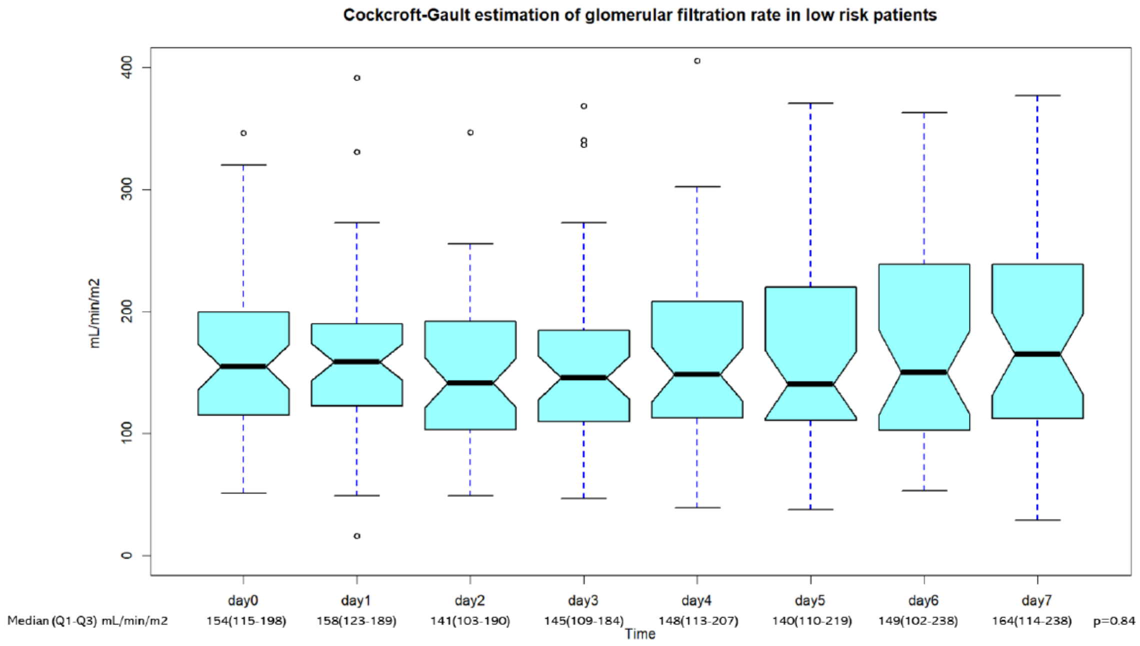

Figure S9. Cockcroft-Gault estimation of glomerular filtration in low risk of AKI patients.

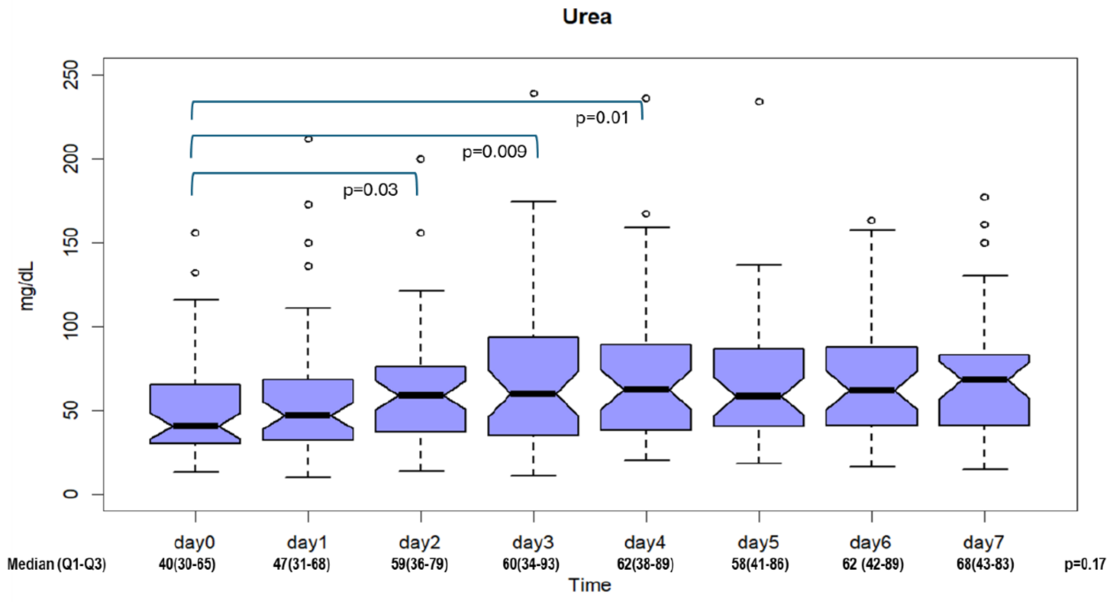

Figure S10. Urea levels during observation period in 52 patients with low risk of AKI at baseline (Day 0).

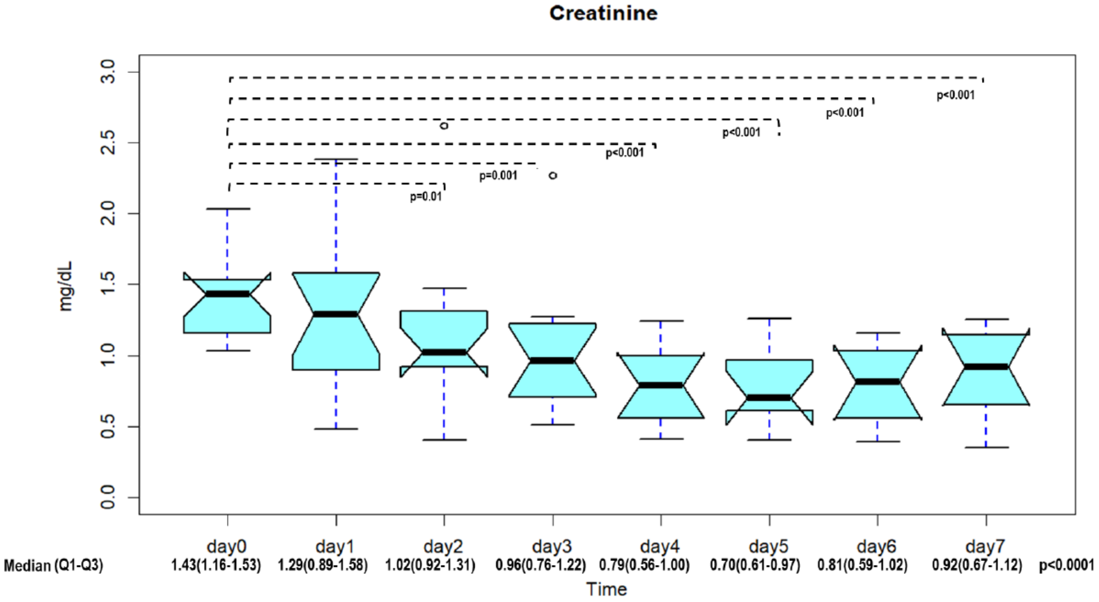

Figure S11. Serum creatinine levels during observation period in 15 patients with high-risk AKI at baseline (Day 0).

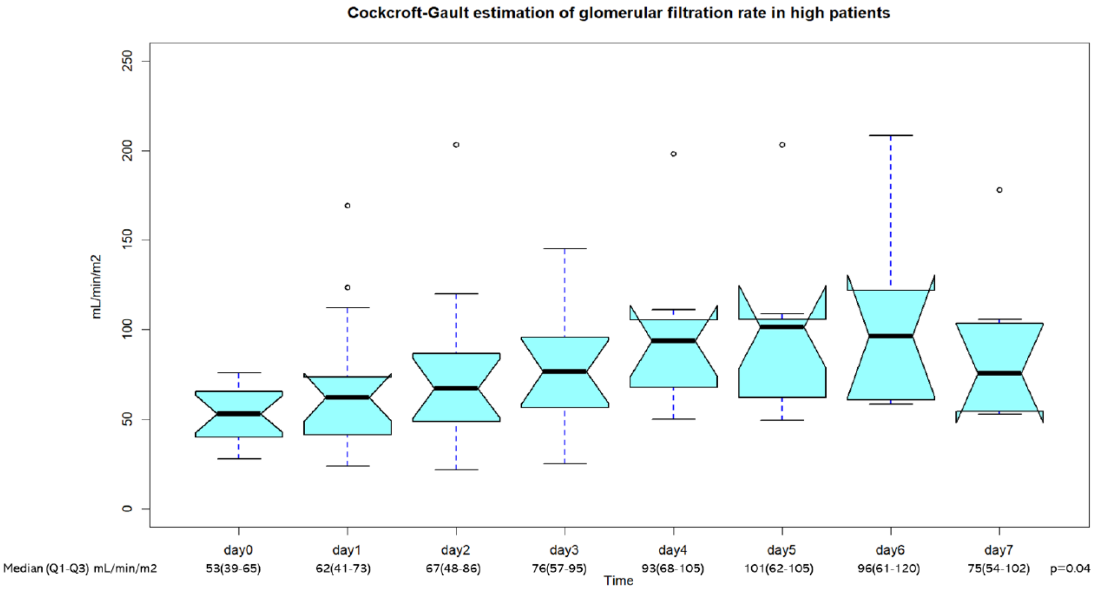

Figure S12. Cockcroft-Gault estimation of glomerular filtration rate in high risk of AKI patients.

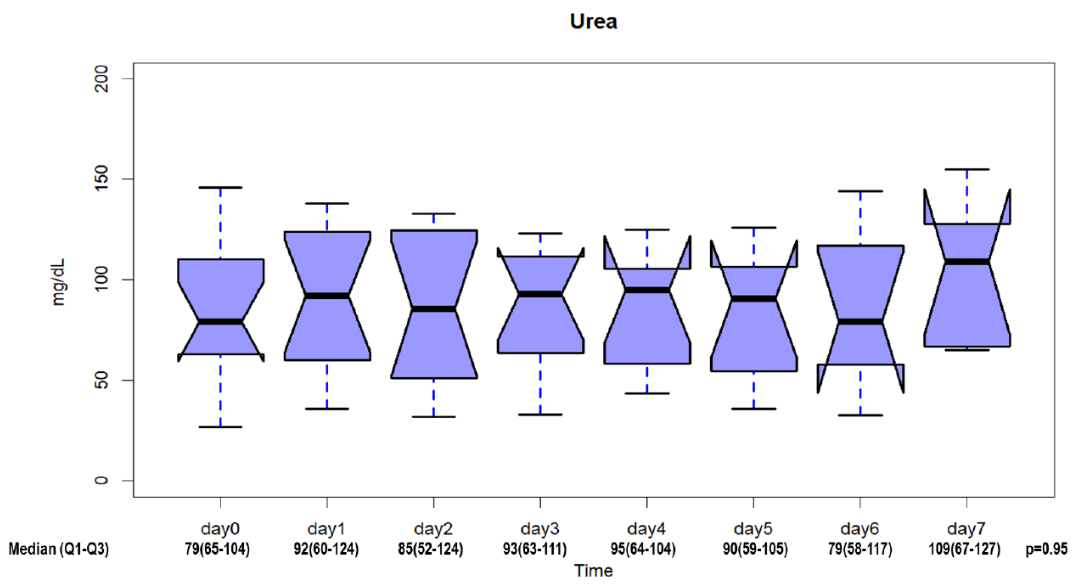

Figure S13. Serum urea levels during observation period in 15 patients with high-risk AKI at baseline (Day 0).

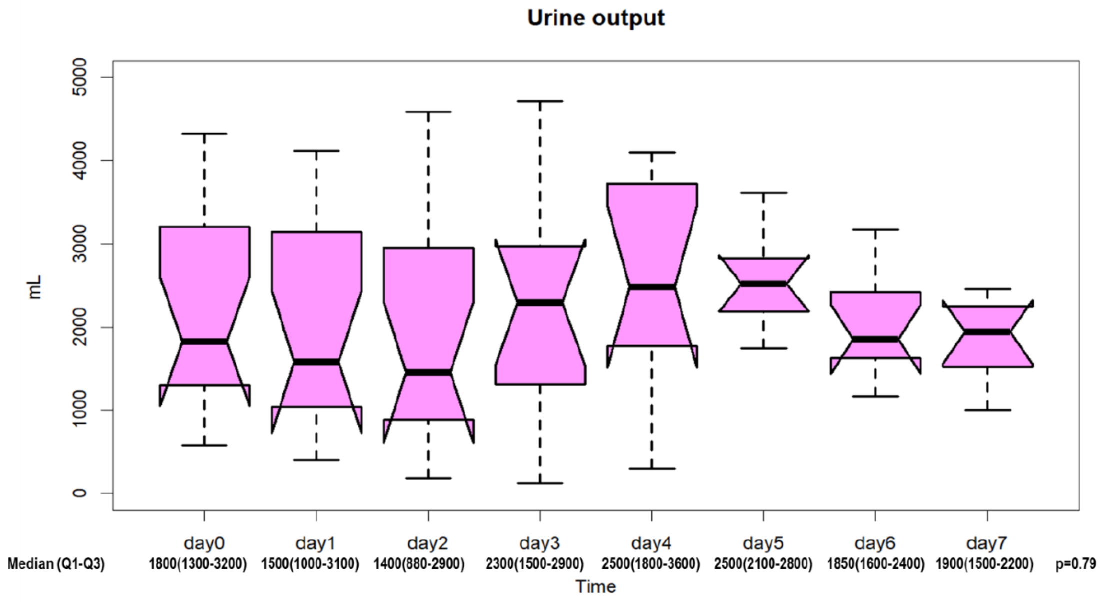

Figure S14. Urine output during observation period in 15 patients with high-risk AKI at baseline (Day 0).

Table S3. Diagnosis of renal dysfunction associated with L-AmB administration according to Acute Kidney Injury Network (AKIN) criteria.

AKIN level	
Grade I	defined as an absolute increase in serum creatinine value ≥ 0.3 mg/dL or an increase between 50% and 100% of the baseline value at 72h.
Grade II	defined as an increase in serum creatinine > 100% (and up to 200%) over baseline at 72h.
Grade III	defined as an increase in serum creatinine > 200% over baseline at 72h, or as an absolute increase in serum creatinine value ≥ 4.0 mg/dL (≥ 354 μmol/L) with an acute increase of at least 0.5 mg/dL (44 μmol/L) or in CRRT.
